# A Tightly Coupled LiDAR-Inertial SLAM for Perceptually Degraded Scenes

**DOI:** 10.3390/s22083063

**Published:** 2022-04-15

**Authors:** Lin Yang, Hongwei Ma, Yan Wang, Jing Xia, Chuanwei Wang

**Affiliations:** 1 School of Mechanical Engineering, Xi’an University of Science and Technology, Xi’an 710054, China; mahw@xust.edu.cn (H.M.); 16105301002@stu.xust.edu.cn (Y.W.); xiajing1984@xust.edu.cn (J.X.); wangchuanwei228@xust.edu.cn (C.W.); 2 Shaanxi Key Laboratory of Mine Electromechanical Equipment Intelligent Monitoring, Xi’an 710054, China

**Keywords:** perceptually degraded scenes, LiDAR, IMU, state estimation, SLAM

## Abstract

Realizing robust six degrees of freedom (6DOF) state estimation and high-performance simultaneous localization and mapping (SLAM) for perceptually degraded scenes (such as underground tunnels, corridors, and roadways) is a challenge in robotics. To solve these problems, we propose a SLAM algorithm based on tightly coupled LiDAR-IMU fusion, which consists of two parts: front end iterative Kalman filtering and back end pose graph optimization. Firstly, on the front end, an iterative Kalman filter is established to construct a tightly coupled LiDAR-Inertial Odometry (LIO). The state propagation process for the a priori position and attitude of a robot, which uses predictions and observations, increases the accuracy of the attitude and enhances the system robustness. Second, on the back end, we deploy a keyframe selection strategy to meet the real-time requirements of large-scale scenes. Moreover, loop detection and ground constraints are added to the tightly coupled framework, thereby further improving the overall accuracy of the 6DOF state estimation. Finally, the performance of the algorithm is verified using a public dataset and the dataset we collected. The experimental results show that for perceptually degraded scenes, compared with existing LiDAR-SLAM algorithms, our proposed algorithm grants the robot higher accuracy, real-time performance and robustness, effectively reducing the cumulative error of the system and ensuring the global consistency of the constructed maps.

## 1. Introduction

SLAM is the basis for autonomous navigation in robots [1], which use various sensors to conduct real-time 6DOF state estimation in 3D space; this state estimation is the key to achieving high-performance SLAM. The LiDAR-based approach can obtain accurate range information for a scene in 3D space in real time and is invariant to ambient lighting conditions. However, due to the problems of motion distortion [2], low-frequency updates, and sparse point clouds [3] in LiDAR measurements, pure LiDAR is unsuitable for robots dealing with aggressive movements or repetitive structures, such as tunnels or narrow corridors. The shortcomings of LiDAR can be compensated for by fusing IMU. Unlike LiDAR, IMU is not affected by drastic changes in structural features or the environment and can provide high-precision pose estimation with high frequency in a short time. However, due to noise and the bias of IMU sensors, error accumulation can drift over time. To overcome these drawbacks of independent sensors, the reliability of state estimation [4,5] can be improved by fusing multiple sensors together. Thus, the robustness of state estimation can be effectively improved through tightly coupled LIO. Many studies have shown that introducing loop detection in SLAM systems can effectively resolve pose drift caused by the accumulation of sensor errors [6,7]. After detecting the loop frame, the pose is continuously optimized to ensure the global consistency of the map, which greatly improves the positioning accuracy.

Despite the superior performance of 3D LiDAR, scenes characterized by single spatial structures, complex environments, uneven illumination [8], narrow spaces and other issues pose new challenges for LiDAR-SLAM: (1) The geometric features in an environment that are recognized by LiDAR (such as points, lines and surfaces) are usually manually defined by mathematical methods, and LiDAR-based solutions can easily degrade when robots operate in environments with no geometric features, repetitive environments and symmetrical structures. (2) The data measured by LiDAR show a high degree of similarity in one dimension, mainly because of the sidewalls in promenade environments, which can provide a lateral localization reference but not a vertical one. In this case, the feature matching of the sidewalls can easily lead to incorrect attitude constraint estimates. (3) Repeated laser features can lead to misleading constraints and attitude drift in the longitudinal direction such that the point cloud alignment converges to the wrong result and the localization performance deteriorates rapidly or fails completely. This severely affects overall SLAM performance and limits the ability of robots to operate autonomously in such environments. At the same time, as the mileage of a robot increases, the trajectory estimated by the odometer will gradually deviate from the real trajectory. To improve the positioning accuracy of the system and the global consistency of the map and suppress the accumulated error of the odometer, when the robot recognizes that the environment is near the pose of a historical frame, relative pose constraints are integrated into the cost function by calculating the relative relationship between the current frame and the loop frame to establish the solution for the overall pose of the system. The main difficulties of loop detection are as follows: (1) Perceived ambiguity. For example, scenes with very similar structures, such as corridors, tunnels and stairs, increase the difficulty of judgment. (2) The sparsity of the point clouds extracted by the LiDAR sensor itself limits the robustness and discrimination of the observation data. (3) The scale of the data can gradually increase the number of frames to be judged as the running time increases, which reduces the real-time performance of the map construction.

Robust 6DOF state estimation and high-performance SLAM can be used to enable robots to more effectively deal with these special perceptually degraded scenes. In this paper, we propose a tightly coupled LiDAR-SLAM fusion algorithm with two sensors, LiDAR and IMU, as input information sources. The framework of the system consists of two parts: front end iterative Kalman filtering and back end pose graph optimization, these are less dependent on external features, have a strong ability to adapt to the environment and are especially suitable for perceptually degraded scenes such as corridors and roadways. However, in perceptually degraded scenes, ensuring the tradeoff between the estimation accuracy, computational complexity and robustness of SLAM systems is still a major problem faced by tightly coupled LIO [9]. Therefore, research in this area is of great importance.

The main contributions of this paper are as follows:

(1) We propose a tightly coupled LiDAR-IMU fusion SLAM algorithm that uses both LiDAR and IMU sensors as input information for perceptually degraded scenes. The results show that our algorithm improves the accuracy, real-time performance and robustness of robots working in such scenarios.
(2) Our algorithm achieves robot state updates by establishing an error-state-based iterative Kalman filter at the front end and adds loop detection and ground constraints in the tightly coupled framework at the back end, which effectively reduces the cumulative error of the system and ensures the global consistency of the constructed maps by optimizing the relative poses between adjacent keyframes.
(3) Our algorithm fuses different types of sensors in a tightly coupled framework to achieve robust 6DOF state estimation and high-performance SLAM for perceptually degraded scenes and outputs motion trajectories, a global map and state estimation in real time after global graph optimization.

The remainder of this paper is organized as follows: In Section 2, we discuss relevant related research. We outline the framework of the system and provide a detailed system overview in Section 3. We describe the pre-processing of the sensor data in preparation for the subsequent work in Section 4. The construction of the front end of the tightly coupled LIO is described in Section 5. We conduct back end pose graph optimization in Section 6. The relevant experiments and results are analyzed in Section 7, followed by conclusions in Section 8.

## 2. Related Work

Of the many studies related to LiDAR-SLAM, we focus on work related to SLAM algorithms for 6DOF state estimation and multi-sensor fusion in robots. The different fusion frameworks of these algorithms are usually classified as loosely coupled and tightly coupled.

### 2.1. LiDAR Odometry and Mapping

LiDAR odometry is usually performed by using scan-matching methods such as ICP [10] and GICP [11] to calculate the relative positional transformation between two consecutive LiDAR frames. Feature-based matching methods have become a popular alternative to matching complete point clouds due to their computational efficiency. For example, in [12], a plane-based real-time LiDAR odometry alignment method was proposed. Assuming an operation in a structured environment, this method extracts planes from a point cloud and matches them by solving a least squares problem. In [13,14], LiDAR odometry was implemented by matching feature points with edge lines and planes using the LOAM algorithm and its improved algorithm, which achieved good results in various scenarios. However, the scanned point cloud is often skewed due to the internal rotation mechanism of the 3D LiDAR and the motion of the sensor. The use of LiDAR alone for attitude estimation is not ideal because using skewed point clouds or features for registration will eventually lead to large drifts. Therefore, LiDAR is often used in conjunction with other sensors, such as cameras or IMUs, for state estimation and SLAM. For example, in [15,16,17,18,19], IMU, LiDAR and GNSS were fused into an extended Kalman filter (EKF) in the optimization phase for robot state estimation.

### 2.2. Loosely Coupled LiDAR-Inertial Odometry

The loosely coupled fusion method usually processes the measurement of each sensor input separately and weights the measurement results to infer the motion state of the robot. LOAM [13] and LeGO-LOAM [14] are loosely coupled systems in which IMU measurements are typically used to mitigate laser degradation in featureless environments, and only the IMU-calculated pose is used as an initial estimate for LiDAR scan alignment and motion distortion correction, not as a constraint for global optimization. The EKF is a commonly used method for loosely coupled fusion. To improve the localization accuracy of autonomous robots, ref. [20] incorporated a wheel speedometer in the loosely coupled LIO framework and established the observation equations of the EKF using the velocity and distance measured by the wheel speedometer. Further, ref. [21] fused LiDAR, IMU, odometry and GNSS based on graph optimization, with the flexible handling of scan matching and joint IMU constraints as well as other observation constraints. In general, loosely coupled methods are computationally efficient [22] and relatively flexible in implementation. However, because these methods ignore the correction of the internal state of the IMU, they are easily disturbed by noise, and the loosely coupled system treats the measurement part as a black box and decouples the measurements of individual sensors; this decoupling leads to information loss [23].

### 2.3. Tightly Coupled LiDAR-Inertial Odometry

Unlike loosely coupled methods, tightly coupled methods typically fuse raw LiDAR feature points (rather than the scanning registration results) with IMU data, which yields a high robustness and accuracy, and can be divided into optimization-based and filter-based methods.

An optimization-based method [7] introduced IMU in the LIO-mapping system pre-integration to minimize the residuals of the IMU and LiDAR measurements; the proposed approach can correct long-term drift through loop detection to achieve good accuracy, but the real-time application of the algorithm is time-consuming. As one of the most advanced algorithms available, LIO-SAM [24] has undergone a large number of experimental tests and outperforms many LIO algorithms that only use LiDAR or are loosely coupled. The sliding window of the LiDAR keyframe is introduced to limit the computational complexity, and the factor graph is used for joint IMU and LiDAR constraint optimization to obtain higher accuracy. This fusion framework is based on a factor graph, which is suitable for multi-sensor fusion. The filter-based method often uses an EKF. However, it is susceptible to linearization errors, which can lead to poor performance and even divergent results [25]. The authors of [6] proposed LINS, which is a robot-centered LiDAR-inertial estimator that recursively corrects the robot state estimation for ground vehicles by using an error state iterative Kalman filter. Although this method has high accuracy and high computational efficiency, it also has drift problems in the long-term navigation process. To reduce the impact of estimation errors caused by linearization, R-LINS [26], FAST-LIO [27] and FAST-LIO2 [28] all use an iterative Kalman filter. Compared with R-LINS and FAST-LIO, FAST-LIO2 uses a new data structure ikd-Tree to save the map; this method can register the original points directly to the map without extracting features, which enables the algorithm to work faster and more accurately.

The most significant difference between our approach and other tightly coupled methods is the specific environment of perceptual degradation. We have chosen a filter-based optimization approach at the front end to improve the system’s robustness, which often uses an extended Kalman Filter (EKF). However, the EKF can easily cause a system to be affected by linearization errors [29]. With regard to the scanning constraints observed by LiDAR, this disadvantage is particularly prominent. If the initial pose is incorrect and leads to the mismatch of extracted features, it is considered to be highly nonlinear. To eliminate the errors caused by incorrect matching, we propose an iterative Kalman filter [30], which can repeatedly find a better match in each iteration. In addition, we use an error state representation to ensure the validity of the linearization [31]. Therefore, our proposed approach constructs an iterative Kalman filter on the front end to fuse LiDAR and IMU measurements to achieve tightly coupled LIO, thus laying the groundwork for back end global optimization studies.

## 3. Overview

### 3.1. System Framework

**Figure 1:**
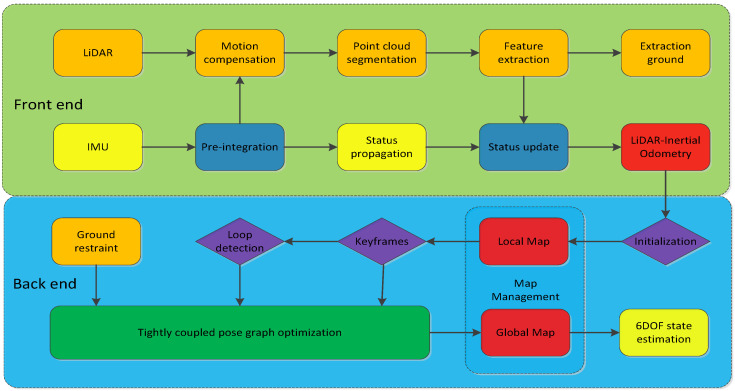
System framework.

Our system receives data from 3D LiDAR and IMU sensors. The goal of the system is for a robot to perform real-time 6DOF state estimation and build a globally consistent map. The system framework of our proposed algorithm, which is shown in Figure 1, is mainly composed of two parts: front end iterative Kalman filtering and back end pose graph optimization. First, pre-processing is conducted on the sensor measurements to compensate for the motion of the original point cloud by pre-integrating the IMU measurements between two consecutive LiDAR frames. Then, stable features are extracted from the corrected point cloud. On the front end (Section 5), the sensor data are pre-processed, the IMU prediction model and the LiDAR observation model are built, the iterative Kalman filter is constructed and the a priori positional attitude of the robot is propagated through a state propagation process, which consists of predictions and observations, to increase the accuracy of the updated pose. Then, the loop is iterated to obtain a tightly coupled LIO. On the back end (Section 6), the keyframe is used to determine if the current pose should be added to the pose graph optimization and loop detection is used to determine if the current position is revisited, which can be expressed as a maximum a posteriori (MAP) problem. In addition, loop detection and ground constraints are added to the tightly coupled framework to optimize the relative pose between adjacent keyframes, reduce cumulative drift, maintain global consistency and improve the positioning accuracy. Finally, information such as the motion trajectory, global map and state estimation are output after pose graph optimization.

### 3.2. Relevant Agreement

To clearly describe the ranging process, we define three reference coordinates. Additionally, we assume that the IMU and robot coordinates are consistent, the origin of the world coordinates is the origin of the robot coordinates when the coordinate is initialized, and the robot coordinates are consistent with the center of the LiDAR and IMU coordinates. Because the connection between the sensors is rigid, the external calibration between the LiDAR and IMU sensors is obtained by offline calibration. The *z*-axis of the world coordinate is defined as coinciding with the normal to the surface of the Earth’s ellipsoid, with the other two axes lying in the plane orthogonal to the *z*-axis, which conforms to the right-hand rule. Table 1 lists the meanings of all variables.

## 4. Sensor Data Pre-Processing

### 4.1. IMU Pre-Integration

The IMU pre-integration algorithm was proposed by Geneva [32]. To avoid continuous propagation of the initial attitude, the world coordinates are separated and only the state change between two consecutive frames in the IMU coordinate is calculated. The position, speed and attitude of two consecutive frames, b_k_ and b_k+1,_ can be expressed as:

(1) $$\begin{array}{l} p_{b_{k+1}}^{b_{k}}=R_{w}^{b_{k}}p_{b_{k+1}}^{w}=R_{w}^{b_{k}}\left(p_{b_{k}}^{w}+v_{b_{k}}^{w}\Delta t_{k}-\frac{1}{2}g^{w}\Delta t_{k}^{2}\right)+\alpha_{b_{k+1}}^{b_{k}}\\ v_{b_{k+1}}^{b_{k}}=R_{w}^{b_{k}}v_{b_{k+1}}^{w}=R_{w}^{b_{k}}\left(v_{b_{k}}^{w}-g^{w}\Delta t_{k}\right)+\beta_{b_{k+1}}^{b_{k}}\\ \gamma_{b_{k+1}}^{b_{k}}={\left[q_{b_{k}}^{w}\right]}^{-1}⊗q_{b_{k+1}^{\prime }}^{w} \end{array}$$

where $\alpha_{b_{k+1}}^{b_{k}}$,$\beta_{b_{k+1}}^{b_{k}}$ and $\gamma_{b_{k+1}}^{b_{k}}$ are the real values of the IMU pre-integration. Therefore, the IMU pre-integration model can be simplified as:

(2) $$\begin{array}{l} \alpha_{b_{k+1}}^{b_{k}}=\iint_{t\in \left[t_{k},t_{k+1}\right]}R\left(\gamma_{t}^{b_{k}}\right)\left({\hat{a}}_{t}-b_{a_{t}}-n_{a}\right)dt^{2}\\ \beta_{b_{k+1}}^{b_{k}}=\int_{t\in \left[t_{k},t_{k+1}\right]}R\left(\gamma_{t}^{b_{k}}\right)\left({\hat{a}}_{t}-b_{a_{t}}-n_{a}\right)dt\\ \gamma_{b_{k+1}}^{b_{k}}=\int_{t\in \left[t_{k},t_{k+1}\right]}[\begin{array}{c} {\frac{1}{2}\left({\hat{\omega }}_{t}-b_{\omega_{t}}-n_{\omega }\right)]}_{R}\gamma_{t}^{b_{k}}dt \end{array} \end{array}$$

### 4.2. Motion Compensation

Due to the rotational mechanism inside the LiDAR sensor, the LiDAR measurements are not synchronized and, therefore, the current frame point cloud is prone to distortion. The LiDAR moves at constant angular and linear velocities at a constant speed. Therefore, the timestamp information of each point can be obtained, a linear interpolation operation used to obtain the pose transformation matrix corresponding to each moment, and the motion compensation of the current frame point cloud can be achieved by using IMU measurements [13,14].

The current frame point cloud is divided into N subframes by $P_{k}$, where $P_{k}^{i}$ is the *i*th subframe of the current laser frame, and *i* ∈ {1, 2,…, N}. Let the pose transformation matrix between two consecutive LiDAR frames in the time interval $\left[t_{k},t\right]$ be $T_{k}^{L}$, which can be obtained by pre-integration of the IMU data and is not explained in detail here. Then, $T_{\left(k,i\right)}^{L}$ is the current *i*th subframe for the LiDAR attitude transformation in the time interval $\left[t_{k},t_{i}\right]$, which can be calculated by the linear interpolation of:

(3) $$T_{\left(k,i\right)}^{L}=\frac{t_{i}-t_{k}}{t-t_{k}}T_{k}^{L}$$

By performing the current subframe transformation, the motion compensated point cloud for $P_{k}^{\prime }$ is obtained as:

(4) $$P_{k}^{\prime }=\left\{T_{\left(k,1\right)}^{L}P_{k}^{1},T_{\left(k,2\right)}^{L}P_{k}^{2},\ldots ,T_{\left(k,i\right)}^{L}P_{k}^{i}\right\},i=1,2,\ldots ,N$$

### 4.3. Feature Extraction

The feature extraction and matching method in this paper is similar to the one introduced in LOAM [13]: the smoothness evaluation variables are first used to select edge points and plane points, and then the corresponding edge line points and plane points in other keyframes are selected and used to construct the residuals of the LiDAR observation model. When a new frame of LiDAR point cloud data is motion compensated, feature extraction is performed. Firstly, by calculating the local smoothness, edge and plane features can be extracted. In addition, the reflectance of a point is used as an additional determinant. If the reflectance of a point is different from the adjacent threshold, it is also considered to be another edge point. Second, the curvature of the points is used to calculate the plane smoothness as an indicator for extraction of the feature information of the current frame. Let *S* be the set of consecutive points *i* returned by the LiDAR in the same scan. Then, the smoothness c of the local surface can be defined and evaluated as:

(5) $$c=\frac{1}{|S|\cdot \left\|X_{\left(k,i\right)}^{L}\right\|}\left\|\sum_{j\in S,j\neq i}\left(X_{\left(k,i\right)}^{L}-X_{\left(k,j\right)}^{L}\right)\right\|$$

The feature points can be divided into two main categories: plane points and edge points. Then, the points in the scan are sorted according to the *c* value and the maximum *c* point is selected as the edge point, that is, the point on the sharp edge in 3D space, which varies greatly in size compared to the surrounding points and has a higher curvature and a higher smoothness. The minimum c point is selected as the plane point, that is, the point on a smooth plane in 3D space, which varies slightly in size compared to the surrounding points and has a lower curvature and a lower smoothness. The LiDAR scanning features of the extracted edge line and plane at time I are expressed as $F_{ei}$ and $F_{pi}$. Then, a set of all extracted features $F_{i}$ in frame *i* is formed, which can be expressed as $F_{i}=\left\{F_{ei},F_{pi}\right\}$.

### 4.4. Extraction Ground

Inspired by [14], adding ground constraints can provide additional constraints on the *z*-axis displacements of keyframe nodes when the ground robot works in different spatial regions while reducing cumulative errors by using more constraint information to improve the pose estimation accuracy. The key to establishing ground constraints is to accurately extract the road planes; determining how to spend the shortest amount of time to complete the planar segmentation and choosing a robust estimation method with few iterations, a high resistance to noise and random sample consensus (RANSAC) are also essential processes [33]. According to the basic principle of RANSAC, a plane is obtained by selecting any three points from each frame of a point cloud. The most commonly used plane equation is ax + by + cz = d, where a a^2^ + b^2^ + c^2^ = 1, d > 0, (a, b, c) is the plane normal vector and d is the distance from the LiDAR sensor to the plane. These four parameters can determine a plane.

Because the robot may travel over uneven ground, it is not possible to construct pose maps with fixed planes as nodes as a constraint. To ensure that the constructed constraints are consistent with the actual situation, a subgraph-based ground extraction method is proposed. Based on the constructed local point cloud map, the true planar parameters are extracted as follows: $\pi_{m}=[n_{a},n_{b},n_{c},d]$. To improve the efficiency of ground extraction, the current position is taken as the origin and the point cloud is searched for in a submap within the radius of the LiDAR measurement distance. Based on the 3D LiDAR attitude at the current moment, the ground parameters are converted to LiDAR coordinates using Equations (6) and (7):

(6) $${\left[n_{a}^{\prime },n_{b}^{\prime },n_{c}^{\prime }\right]}^{T}=R_{t}\cdot {\left[n_{a},n_{b},n_{c}\right]}^{T}$$

(7) $$d^{\prime }=d-t_{t}\cdot {\left[n_{a}^{\prime },n_{b}^{\prime },n_{c}^{\prime }\right]}^{T}$$

where $\pi \prime_{m}=[n\prime_{a},n\prime_{b},n\prime_{c},d]$ denotes the coordinates of the ground in the LiDAR coordinate and $\left[R_{t},t_{t}\right]$ is the positional attitude of the LiDAR at moment *t*.

The calculation of the error between the attitude node and the ground plane node [34] can be expressed as:

(8) $$e_{i,m}=q\left(\pi_{m}^{\prime }\right)-q\left(\pi_{t}\right)$$

(9) $$q\left(\pi \right)=\left[\arctan(\frac{n_{a}}{n_{b}}),\arctan(\frac{n_{c}}{\left|n\right|}),d\right]$$

## 5. Front End: Tightly Coupled LiDAR-Inertial Odometry

Tightly coupled LIO uses IMU measurements and features extracted by LiDAR in two consecutive scans to estimate the relative positional transformation of the robot. To avoid large linearization errors caused by growing uncertainty due to IMU measurements [35], we constructed a robot-centric iterative Kalman filter.

### 5.1. State Definitions

To facilitate the derivation of the iterative Kalman filter, we define the state $x_{b_{k+1}}^{b_{k}}$ from time *k* to *k* + 1 under the IMU coordinate, expressed as:

(10) $$x_{b_{k+1}}^{b_{k}}:=\left[p_{b_{k+1}}^{b_{k}},v_{b_{k+1}}^{b_{k}},q_{b_{k+1}}^{b_{k}},b_{a},b_{g},g^{b_{k}}\right]$$

According to the basic principle of the ESKF [36], once the error state δx is solved, we add the error state to the a priori state ${\bar{x}}_{b_{k+1}}^{b_{k}}$ to obtain the a posteriori state $x_{b_{k+1}}^{b_{k}}$, which can be expressed as:

(11) $$x_{b_{k+1}}^{b_{k}}={\bar{x}}_{b_{k+1}}^{b_{k}}⊕\delta x=\left[\begin{array}{c} {\bar{p}}_{b_{k+1}}^{b_{k}}+\delta p\\ {\bar{v}}_{b_{k+1}}^{b_{k}}+\delta v\\ {\bar{q}}_{b_{k+1}}^{b_{k}}⊗\exp(\delta \theta )\\ {\bar{b}}_{a}+\delta b_{a}\\ {\bar{b}}_{g}+\delta b_{g}\\ {\bar{g}}^{b_{k}}+\delta g \end{array}\right]$$

where ⊗ is the product of quaternions, and exp(·) represents the Euler angles to quaternions.

### 5.2. Definition of the Motion Equation

The equation of motion over linearized continuous time is defined as:

(12) $$\delta \hat{x}(t)=F_{t}\delta x(t)+G_{t}w$$

where w is the Gaussian noise vector $w={\left[n_{a}^{T},n_{g}^{T},n_{b_{a}}^{T},n_{b_{g}}^{T}\right]}^{T}$, $F_{t}$ is the error state transfer matrix at moment *t* and $G_{t}$ is the noise Jacobi matrix at moment *t*.

(13) $$F_{t}=\left[\begin{array}{cccccc} 0 & I_{3} & 0 & 0 & 0 & 0\\ 0 & 0 & -R_{t}^{b_{k}}{\left[{\hat{\alpha }}_{t}\right]}_{\times } & -R_{t}^{b_{k}} & 0 & -I_{3}\\ 0 & 0 & -{\left[{\hat{\omega }}_{t}\right]}_{\times } & 0 & -I_{3} & 0\\ 0 & 0 & 0 & 0 & 0 & 0\\ 0 & 0 & 0 & 0 & 0 & 0\\ 0 & 0 & 0 & 0 & 0 & 0 \end{array}\right]$$

(14) $$G_{t}=\left[\begin{array}{cccc} 0 & 0 & 0 & 0\\ -R_{t}^{b_{k}} & 0 & 0 & 0\\ 0 & -I_{3} & 0 & 0\\ 0 & 0 & I_{3} & 0\\ 0 & 0 & 0 & I_{3}\\ 0 & 0 & 0 & 0 \end{array}\right]$$

where ${\left[\cdot \right]}_{\times }\in {\mathbb{R}}^{3\times 3}$ represents the conversion of a 3D vector to a skew-symmetric matrix. $I_{3}$ is the unit matrix. ${\hat{a}}_{t}$ is the true value of the acceleration at moment *t* and ${\hat{\omega }}_{t}$ is the true value of the angular velocity at moment *t*.

Therefore, the equation of motion linearized in continuous time by Equation (12) is discretized [37] to obtain the mean value of the prior state via Equation (15) and the variance via Equation (16), respectively.

(15) $$\delta x_{i+1}=\left(I+F_{i}\Delta t\right)\delta x_{i}$$

(16) $$P_{i+1}^{b_{k}}=\left(I+F_{i}\delta t\right)P_{i}^{b_{k}}{\left(I+F_{i}\delta t\right)}^{T}+\left(V_{i}\delta t\right)Q{\left(V_{i}\delta t\right)}^{T}$$

where P is the covariance matrix of δx. Q is the covariance matrix of w, which is obtained offline by sensor calibration.

### 5.3. Definition of the Observation Equation

The observation model is mainly obtained by matching the features extracted by the LiDAR to the point cloud and is defined as:

(17) $$\delta \hat{y}(t)=f{\left({\bar{x}}_{b_{k+1}}^{b_{k}}⊕\delta x\right)}_{{\left(J_{k}M_{k}J_{k}^{T}\right)}^{-1}}$$

where $J_{k}$ is the measurement noise of f(·), and $M_{k}$ is the covariance matrix of the measurement noise.f(·) represents a stacked residual vector computed from point-to-edge pairs or point-to-plane pairs.

Given the error term in $x_{b_{k+1}}^{b_{k}}$, f(·) corresponds to $p_{i}^{l_{k+1}}$ and the *i*th characteristic point in the LiDAR coordinate *k* + 1 frames, and the observation equation can be described as

(18) $$f_{i}\left(x_{b_{k+1}}^{b_{k}}\right)=\{\begin{array}{ll} \frac{\left|\left({\hat{p}}_{i}^{l_{k}}-p_{a}^{l_{k}}\right)\times \left({\hat{p}}_{i}^{l_{k}}-p_{b}^{l_{k}}\right)\right|}{\left|p_{a}^{l_{k}}-p_{b}^{l_{k}}\right|} & \;\mathrm{if}\;p_{i}^{l_{k+1}}\in F_{e}\\ \frac{\left|{\left({\hat{p}}_{i}^{l}-p_{a}^{l_{k}}\right)}^{T}\left(\left(p_{a}^{l_{k}}-p_{b}^{l_{k}}\right)\times \left(p_{a}^{l_{k}}-p_{c}^{l_{k}}\right)\right)\right|}{\left|\left(p_{a}^{l_{k}}-p_{b}^{l_{k}}\right)\times \left(p_{a}^{l_{k}}-p_{c}^{l_{k}}\right)\right|} & \;\mathrm{if}\;p_{i}^{l_{k+1}}\in F_{p} \end{array}$$

(19) $${\hat{p}}_{i}^{l_{k}}=R_{l}^{b^{T}}\left(R_{b_{k+1}}^{b_{k}}\left(R_{l}^{b}p_{i}^{l_{k+1}}+p_{l}^{b}\right)+p_{b_{k+1}}^{b_{k}}-p_{l}^{b}\right)$$

where ${\hat{p}}_{i}^{l_{k}}$ is the pose transformation of the point $p_{i}^{l_{k+1}}$ from the *k*+1th frame to the *k*th frame. For edge points, the distance is between point $p_{i}^{l_{k+1}}$ and the line formed by $p_{a}^{l_{k}}p_{b}^{l_{k}}$. For planar points, the distance is between point $p_{i}^{l_{k+1}}$ and the plane formed by $p_{a}^{l_{k}}p_{b}^{l_{k}}p_{c}^{l_{k}}$.

### 5.4. Status Update

In this paper, an iterative extended Kalman filter (IEKF) is used and the state update can be abstracted as an optimization problem. Because the error state is composed of the deviation of the prior state and the residual of the observation model, the error state update problem can be turned into a joint optimization problem of solving for the minimum of the deviation of the prior state and the minimum of the deviation of the observation model relative to the pose solution, which can be expressed as:

(20) $$r_{S}(x)\underset{}{=\min}{\left\|\delta x\right\|}_{P{\left(k\right)}^{-1}}+{\left\|f({\bar{x}}_{b_{k+1}}^{b_{k}}⊕\delta x)\right\|}_{{\left(J_{k}M_{k}J_{k}^{T}\right)}^{-1}}$$

where ‖·‖ denotes the M-norm.

Thus, the Kalman gain equation for the ESKF iterative observation can be expressed as Equation (21), and the mean value of the posterior state can be expressed as Equation (22):

(21) $$K_{k,j}=P_{k}H_{k,j}^{T}{\left(H_{k,j}P_{k}H_{k,j}^{T}+J_{k,j}M_{k}J_{k,j}^{T}\right)}^{-1}$$

(22) $$\delta x_{j+1}=\delta x_{j}+K_{k,j}\left(H_{k,j}\delta x_{j}-f\left({\bar{x}}_{b_{k+1}}^{b_{k}}⊕\delta x_{j+1}\right)\right)$$

where $H_{k,j}$ is the Jacobi matrix of $f\left({\bar{x}}_{b_{k+1}}^{b_{k}}⊕\delta x_{j+1}\right)$ and $\Delta x_{j}$ represents the correction vector at the *j*th iteration. Note that in each iteration, new matching edges and matching surfaces are found to further minimize the metric error, so $H_{k,j}$, $J_{k,j}$ and $K_{k,j}$ are recomputed.

When $f\left(x_{b_{k+1}}^{b_{k}}\right)$ is below a certain threshold, $P_{k}$ is updated using Equation (23), which can be obtained after the *n*th iteration, and the variance of the posterior state can be expressed as:

(23) $$P_{k+1}=\left(I-K_{k,n}H_{k,n}\right)P_{k}{\left(I-K_{k,n}H_{k,n}\right)}^{T}+K_{k,n}M_{k}K_{k,n}^{T}$$

By iterative updates, the exact posterior state $x_{b_{k+1}}^{b_{k}}$ can be obtained, and this information can be used as the prior state at the next moment, which is in turn set as the prior state at the next moment.

### 5.5. State Composition

The positional information was calculated earlier under the robot-centered reference coordinate. Therefore, to obtain global coordinate information, state synthesis is needed, and the synthesis formula can be expressed as:

(24) $$x_{w}^{b_{k+1}}=\left[\begin{array}{c} p_{w}^{b_{k+1}}\\ q_{w}^{b_{k+1}} \end{array}\right]=\left[\begin{array}{c} R_{b_{k}}^{b_{k+1}}\left(p_{w}^{b_{k}}-p_{b_{k+1}}^{b_{k}}\right)\\ q_{b_{k}}^{b_{k+1}}⊗q_{w}^{b_{k}} \end{array}\right]$$

## 6. Back End: Pose Graph Optimization

### 6.1. Initialization

LiDAR and IMU sensors are used, where the acceleration deviation $b_{a_{0}}$ of the IMU and the deviation $b_{\omega_{0}}$ of the gyroscope can be obtained by offline calibration. The external calibration matrix $R_{l}^{b}$, $p_{l}^{b}$ between the LiDAR and the IMU can be obtained by offline calibration. The initial roll and pitch of the global poses are obtained by unbiased acceleration measurements before the motion. The initial local gravity is obtained from the initial roll and pitch by converting the gravity vector represented in the navigation frame to the current local frame.

### 6.2. Keyframe Selection

To keep a limited number of poses for estimation, on the back end, we deploy a keyframe selection strategy. The selection of keyframes can greatly improve the computational efficiency and ensure that the algorithm can run in real time on larger environmental maps.

When selecting keyframes, matching errors and redundant keyframes must be reduced to decrease the computational effort, while sparse keyframes increase the uncertainty of interframe observations and degrade the map quality. Usually, the first point cloud is used as the keyframe, and the keyframe selection criteria based on the real-time point cloud are based on the relative positional change between consecutive frames. Let the pose of the *k*−1th keyframe be *T_k_*_−1_ and the pose of the *k*th keyframe be *T_k_* [38]. The relative positional change of consecutive keyframes can be obtained using Equation (25):

(25) $$\Delta T_{k-1,k}=T_{k-1}^{-1}T_{k}=\left(\begin{array}{cc} \Delta r & \Delta s\\ 0 & 1 \end{array}\right)$$

where the rotation of the change between two consecutive keyframes can be expressed as ΔR, the translation of the change between two consecutive keyframes can be expressed as Δs and the time of the transformation between two consecutive keyframes can be expressed as Δt.

(26) $$\Delta R=\arccos\left(\frac{\mathrm{trace}(\Delta r)-1}{2}\right)$$

(27) $$\left\|\Delta s\right\|=\sqrt{\Delta x^{2}+\Delta y^{2}+\Delta z^{2}}$$

When any of the above three criteria exceed the set threshold, the current kth frame of the point cloud is selected as a keyframe. The other LiDAR frames between the two keyframes are discarded. Adding keyframes in this way achieves not only a balance between map density and memory consumption but also a uniform distribution in space, which is suitable for real-time nonlinear optimization.

### 6.3. Mapping

When a point cloud frame is detected as a keyframe, the environment map of the current frame needs to be updated, and the main method is to convert the real-time point cloud information of the keyframe to the world coordinates by coordinate transformation [39]. When *k* + 1 frames are selected as sub-keyframes, continuous sub-keyframe optimization is performed using ICP and the relative change in pose between sub-keyframes is $T_{k+1}^{L}$. By the relative change of the *k*th sub-keyframe to the world coordinates as $T_{k}^{W}$, the relative change of the *k*+1th sub-keyframe to the world coordinate can be obtained as $T_{k+1}^{W}=T_{k}^{W}T_{k+1}^{L}$. Finally, the point cloud coordinates of the *k* + 1 sub-keyframes are converted into the world coordinates, and the converted sub-keyframes are merged into a voxel mapping $M_{i}$. Because two types of features are extracted in the previous feature extraction step, *M_i_* consists of two sub-voxel maps, that is, the edge feature voxel map set $M_{i}^{e}$ and the planar feature voxel map set $M_{i}^{p}$. The interrelationship between the LiDAR frames and the voxel maps is as follows:

(28) $$M_{i}=\left\{M_{i}^{e},M_{i}^{p}\right\}$$

where $M_{i}^{e}=\prime F_{i}^{e}\cup \prime F_{i-1}^{e}\cup \ldots \cup \prime F_{i-n}^{e}$,$M_{i}^{p}=\prime F_{i}^{p}\cup \prime F_{i-1}^{p}\cup \ldots \cup \prime F_{i-n}^{p}$. $\prime F_{i}^{e}$ and $\prime F_{i}^{p}$ are the edge line and plane features, respectively, extracted by the desired transformation sub-keyframe that are down-sampled to eliminate duplicate features that fall in the same pixel cell, while the point cloud of the current sub-keyframe is created as a local map and updated by an octree structure.

### 6.4. Loop Detection

To reduce the cumulative error of the sensor, we add loop detection. When the robot detects all historical keyframes during operation, pose graph optimization is combined with all relevant positions and poses in the historical keyframes. When performing loop detection, the current real-time keyframe is first compared with the historical keyframe, and then the candidate loop frame is selected. The following conditions are met: (1) the index of the current keyframe is larger than the index of the history keyframe; (2) the difference between the track distance of the current keyframe and the history keyframe is larger than the set threshold; and (3) the relative translation distance between the current keyframe and the history keyframe is smaller than the set threshold. Finally, the candidate loop frame with the highest score is selected by comparing the candidate loop frames and the currently registered keyframes. The keyframes and candidate loop frames are added as nodes to the SLAM pose graph optimization, and the edge constraints are the relative poses obtained from point cloud registration.

The loop detection algorithm [40] is described in Algorithm 1. Suppose the current frame point cloud is represented as *P_m_* in the *B* coordinates and its relative pose change to the world coordinates is *T_m_*. If the keyframe database contains the keyframe pose set *D_T_* and the point cloud set *D_P_*, then a kd tree search with radius r is performed. Therefore, the problem to be solved is to show that the loop has been detected if we can find $T\prime_{m}$ in the keyframe pose set *D_T_* such that the sum of the root mean square error of the point cloud *P_m_* of the current frame after the relative change $T_{ICP}$ with the nearest point in D_P_ is minimized. The keyframe pose *D_T_* registers the keyframe point cloud D_P_ to the local point cloud map M constructed in the world coordinate space.
**Algorithm 1** Loop Detection Algorithm.Input: $P_{m}$ and $T_{m}$ from the current frame point cloudOutput: $T_{ICP}$1: if ($T_{m}$,$P_{m}$) is Keyframe() then2:      if $D_{T}\neq \phi$ or $D_{P}\neq \phi$ then3:           $T\prime_{m}$← KDtree.RadiusSearch($T_{m}$,$D_{T}$,r);4:            M ← registerPointCloud($D_{T}$,$D_{P}$);5:            if $T\prime_{m}\neq 0$ then6:                 $T_{ICP}$← ComputeICP($P_{m}$, M,$T_{m}$);7:         end if8:    end if9:  $T\prime_{m}$ ← 010:   $D_{T}=D_{T}\cup T_{m}$ and $D_{P}=D_{P}\cup P_{m}$11: end if 

Only the current pose of the point cloud in the world coordinates for the current frame is obtained, and *T_ICP_* and *M* are fed to the pose graph optimization for state correction. The state correction can be expressed as a modification of Equation (29) by solving the following cost function:

(29) $$r_{M}\left({\hat{z}}_{\left(j,m\right)},\chi \right)={\bar{P}}_{j}^{w}-{\bar{P}}_{m}^{w}=R_{b_{k}}^{w}\left(R_{l}^{b}{\bar{P}}_{j}^{l_{k}}+p_{l}^{b}\right)+p_{b_{k}}^{w}-{\bar{P}}_{m}^{w}$$

where ${\bar{P}}_{m}^{w}\in M$ is the closest point to ${\bar{P}}_{j}^{w}$ in the global map. By solving the modified cost function, the current state can be used for repositioning in the global map.

### 6.5. Pose Graph Optimization Construction

To effectively solve the large-scale simultaneous localization and map building problem, the method of cluster adjustment (BA) is often used; that is, the map optimization uses sensor poses and spatial points. However, as the trajectory of the robot increases, the BA method decreases the computational efficiency of the system as the size of the map continues to increase. To address this issue, the pose graph optimization only uses the trajectory as a constraint for the pose estimation and no longer optimizes the pose estimation of the feature points. Based on bitmap optimization theory, the keyframe pose, ground constraint and loop detection constraint are calculated to construct a multi-constraint-based cost function for bitmap optimization.

The pose graph optimization problem can be efficiently solved by standard optimization methods such as the Gauss–Newton or Levenberg–Marquardt (LM) algorithms [41]. This problem has also been integrated into the Generalized Graph Optimization (G2O) of the Optimization Library [42]. Our goal is to estimate the 6DOF self-motion of the robot and simultaneously build a globally consistent map. Assuming that these measurement errors conform to a zero-mean Gaussian distribution, solving the maximum a posteriori (MAP) problem is equivalent to minimizing the negative log-likelihood, which can be written in the form of the squared Marxian distance:

(30) $${\hat{X}}_{n}=\underset{}{\arg\min}\left\|r_{S}(x)\right\|+\sum \left\|e_{i,m}\right\|+\sum \left\|r_{M}\left({\hat{z}}_{\left(j,m\right)},\chi \right)\right\|$$

where $r_{S}(x)$ denotes the deviation of the error state estimated by LIO,$e_{i,m}$ denotes the error between the attitude node and the ground plane node and $r_{M}\left({\hat{z}}_{\left(j,m\right)},\chi \right)$ denotes the deviation of the current state of the loop detection in the global map.

## 7. Tests and Analyses

**Figure 2:**
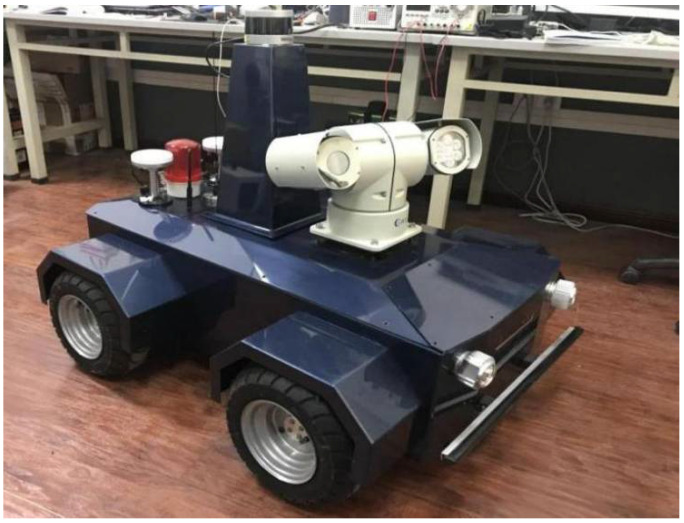
Wheeled inspection robot.

We conducted a series of quantitative and qualitative analysis experiments on the performance of the proposed tightly coupled LiDAR-IMU fusion SLAM algorithm and compared the results with those of other state-of-the-art LiDAR-SLAM methods. All methods were tested under the same conditions. The hardware platform was an inspection robot with sensors and an on-board computer, as shown in Figure 2. The sensor consisted of a LiDAR (Velodyne VLP-16) with a sampling frequency of 10 Hz and an IMU (hipnuc-CH110) with a sampling frequency of 200 Hz. The on-board computer was an Intel Core i7 with a 2.7 GHz clock, eight cores and 16 G of RAM. All algorithms were implemented in C++ and executed on an Ubuntu 18.04 system using the medoic version of ROS.

### 7.1. Implementation and Datasets

Since accurate true values are not available for comparison for perceptually degraded scenes, in order to validate the performance of our proposed method (including accuracy, cumulative error and robustness) while ensuring the experimental soundness, we first used public dataset 1 and public dataset 2 for quantitative analysis. Note that all algorithms were tested without the loop module. To verify whether our proposed method causes the accuracy of the SLAM system to decrease as the speed of motion changes, we used the public dataset 1, which is a six-set fast-to-slow dataset comparing rotation and translation errors, whose true values are provided by the indoor high-precision motion capture instrument. To verify whether our proposed method increases the cumulative error of the system as it operates for a long time, we used public dataset 2, which is the 2011_09_30_drive_0028 sequence of the KITTI datasets, whose true values are provided by outdoor high-precision GPS. Subsequently, to verify whether our proposed method is robust under three different real perceptual degradation environments, we recorded three self-picked datasets with our own experimental platform, and the self-picked datasets were qualitatively analyzed for real promenade environments under three perceptual degradation scenes. Furthermore, we compared the method with the a_loam, lego_loam and lio_sam methods. We verified the excellent robustness of our proposed algorithm through horizontal comparison between different algorithms and vertical comparison between different scenes. In addition, we designed ablation experiments to verify the effects of adding ground constraints and loop detection constraints on the overall optimization results. Finally, a runtime analysis was performed for the main modules.

### 7.2. Quantitative Analysis

#### 7.2.1. Tests at Different Movement Speeds

Table 2 shows the root mean square error (RMSE) results for different speeds and methods. LOAM [1] was considered as the baseline. LIO represents the local window optimized odometry method, LIO-raw is the result obtained by removing motion compensation on top of LIO and LIO-no-ext is the result obtained by removing online external parameter estimation on top of LIO. The “ours” column represents the proposed LIO generated by the iterative Kalman filter. The best results are shown in bold.

The results show that our proposed algorithm always provided more accurate translational and rotational states for both fast and slow motions. The table also shows that, with motion compensation and online external reference estimation, LIO provided a better performance, especially in the case of fast motion, probably because more IMU excitation had to be generated. The proposed method, in contrast, was not affected by the speed of motion and performed better at any speed of motion relative to LIO.

#### 7.2.2. Deviation Test with Long Time Change

**Figure 3:**
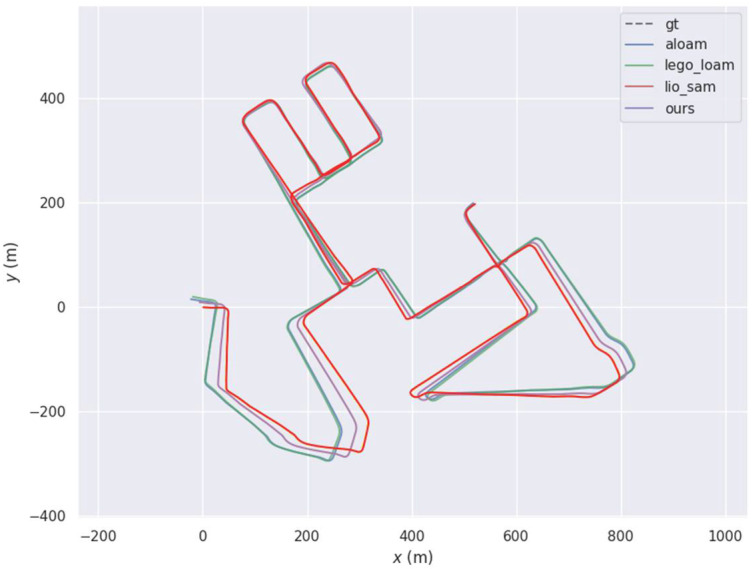
Comparison between the motion trajectories generated by the four different algorithms and the true values.

**Figure 4:**
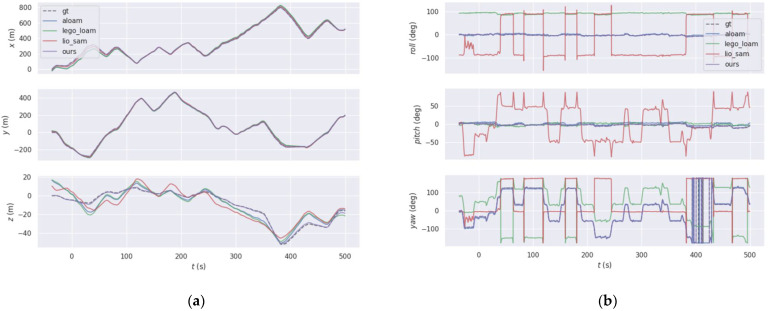
Comparison between the trajectory error and the true value produced by the four different, they should be listed as: (**a**) translation error; (**b**) rotation error.

To evaluate how the error varied over time, we used the 2011_09_30_drive_0028 sequence from the KITTI datasets, with the four different algorithms producing roughly the same motion trajectory but with subtle differences in some areas. To explore the differences between the algorithms, the EVO tool was used for quantitative analysis. The final motion trajectories of the different methods are shown in Figure 3 and the trajectory error and the true value produced by the four different methods are shown in Figure 4.

To further refine the comparison experiments, we analyzed the maximum deviation, mean deviation, minimum deviation, root mean square error and standard deviation of the KITTI datasets 2011_09_30_drive_0028 sequence for the four different algorithms for each relative translation and rotation, as shown in Table 3. The best results are shown in bold.

The statistical results show that the proposed algorithm had smaller translation and rotation errors than the other three algorithms when tested on the same datasets. Therefore, the accuracy of the state estimation of our proposed algorithm is better than that of the other methods.

### 7.3. Qualitative Analysis

**Figure 5:**
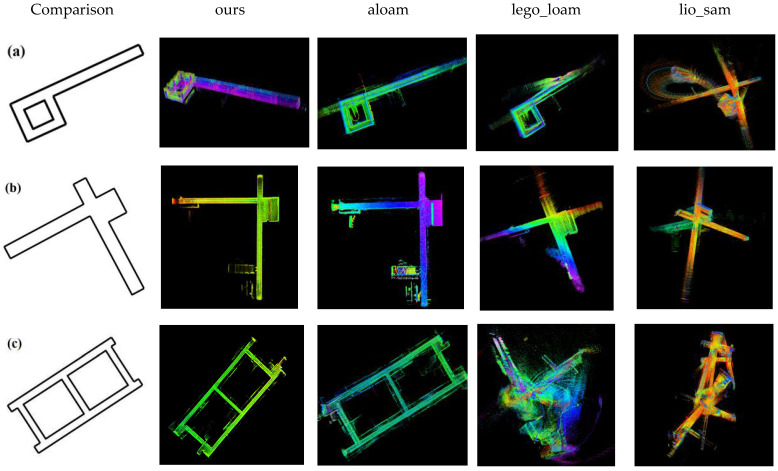
The point cloud map was constructed by four SLAM methods in the current experimental environment, they should be listed as: (**a**) Corridor scene on the 3rd floor of the mechanical building; (**b**) corridor scene on the 21st floor of the innovation building; (**c**) corridor scene on the 11th floor of the science and technology building.

We conducted three experiments with the same sensor configuration in different test environments to verify the performance of the proposed algorithm for perceptually degraded scenes. The datasets included corridor scene 1 on the 3rd floor of the mechanical building, corridor scene 2 on the 21st floor of the innovation building and corridor scene 3 on the 11th floor of the science and technology building. The proposed algorithm was analyzed and compared with the aloam, lego_loam and lio_sam methods, and its point cloud map was constructed in the current experimental environment, as shown in Figure 5.

As shown in Figure 5, four different SLAM algorithms were used to construct point cloud maps under three perceptually degraded scenes. The differences between the above three different scenes are: Figure 5a,b scenes do not differ much in form, only in specific details, and the “8-letter” structure of Figure 5c is a loop detection structure. The similarity is that since all three test scenes are all perceptually degraded scenes, their point clouds have relatively few features, thus posing a great challenge to the proposed algorithm. For the four different algorithms, the point cloud maps constructed by the proposed method showed an excellent performance in terms of completeness and the realism of the geometric structure in all three scenarios. While aloam also performed well in all three scenes, a small amount of mismatching of the point cloud was caused by the occurrence of successive similar rotations and translations in scene a. Lego_loam constructed a complete point cloud map for the rough outline of the corridor in both scenarios a and b. However, the structure of the generated point cloud map was distorted and incomplete due to the drift on the *Z*-axis. In the c scene, the structural symmetry in the annular scene was too similar, resulting in interference of the extracted features of the original point cloud data by a large number of similar point cloud features, and the entire SLAM system failed. Lio_sam constructed the geometric structure of the surrounding environment in a short period of time for scenarios a, b and c. However, as the mileage of the laser odometer increased, when the system detected the loop, the point cloud map construction failed because the scenarios were highly similar, resulting in the establishment of an incorrect loop detection constraint.

In conclusion, the four different algorithms build different point cloud maps in the same environment because of their strengths and weaknesses. The scheme of aloam adopts the assumption of uniform motion model and uses IMU data for distortion correction of point clouds. The system has good real-time performance, but there is no loop detection module, which causes it to drift in large-scale tests. The lego_loam method is an optimized version of loam which segments and clusters the raw data frames scanned by LiDAR, labels the ground point cloud, and improves the accuracy of state estimation by two-step LM optimization. However, because it relies too much on the extraction of point surface point clouds, once the ground point cloud extraction fails, the state estimation error will increase. Lio_sam is an extended version of lego_loam which solves the least-squares problem by continuously adding various factors. However, loop detection requires a high correlation of correct data, and incorrect matching can lead to the failure of the whole SLAM system. The method we propose incorporates the advantages of all the above algorithms. First, the front end adopts the ideas of homogeneous model assumption from loam and extraction of ground features from lego_loam, respectively. However, we improve the Kalman filter model in LINS by the RANSAC method for point cloud ground extraction and propose an iterative Kalman filter model to eliminate the error caused by mismatching in order to achieve a tightly coupled LiDAR-inertial odometry. Secondly, the back end draws on the idea of factor graph optimization in lio_sam to construct a cost function for bit-pose graph optimization by adding ground constraints and loop detection constraints through the selection strategy of keyframes and further improve the accuracy of state estimation by solving the maximum a posteriori problem. Therefore, our proposed method performs well under three different perceptual degradation environments.

### 7.4. Ablation Experiments

To further analyze the localization and map-building effects of the proposed algorithm, the effects of the ground constraint and loop detection back end pose graph optimization modules were analyzed separately.

#### 7.4.1. Effect of Ground Restraint

**Figure 6:**
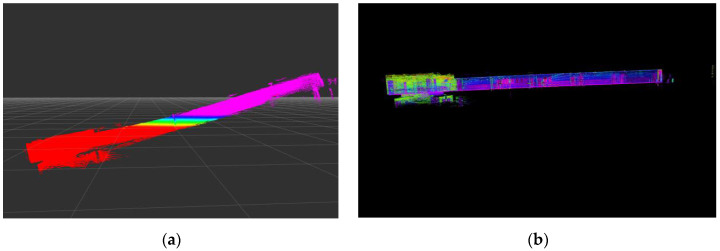
Effect of ground constraints on positioning and map building, they should be listed as: (**a**) Without ground constraint, (**b**) with ground constraint.

The results of the experimental analysis show that the ground constraint of the back end pose graph optimization in the system had a great influence on the map building results, as shown in Figure 6. Figure 6a shows that without adding ground constraints in the back end pose graph optimization, the constructed point cloud map could not be corrected, which tilted it to a certain angle. Figure 6b shows the addition of the ground constraint in the back end pose graph optimization to correct the *Z*-axis drift during the positioning and map-building process and the construction of a globally consistent point cloud map.

#### 7.4.2. Effect of Loop Detection

**Figure 7:**
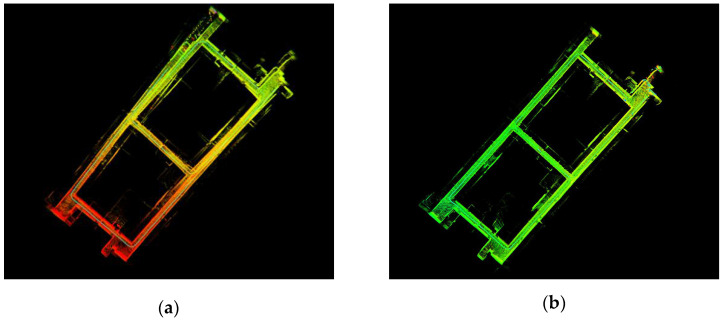
Effect of loop detection on localization and map building: (**a**) Without loop detection and (**b**) with loop detection.

The loop detection of the back end pose graph optimization in the system had a substantial impact on the construction of the map, as shown in Figure 7. Figure 7a shows that no loop constraint was used in the back end pose graph optimization, which led to a large deviation in the constructed point cloud map, such that part of the map was overshadowed in some areas in the upper left corner. Figure 7b shows the addition of the loop constraint in the back end pose graph optimization. The cumulative error of the global map was corrected, and finally, a globally consistent point cloud map was constructed.

### 7.5. Runtime Analysis

To explore the real-time performance of the proposed algorithm, we conducted experiments on the self-selected dataset and the KITTI dataset and analyzed the running time of the main modules for the down-sampling, pre-processing and scan-matching front end steps; on the back end, the running time was analyzed for the map construction, loop detection and pose graph optimization steps. The details are shown in Table 4. As the table shows, because our self-selected dataset consisted of VLP-16 data and the KITTI dataset consisted of HDL-64 data, more time was required to process the individual modules of the KITTI dataset with the same sampling frequency for the LiDAR-SLAM system. We analyzed the self-selected data, and for the front end LiDAR point cloud and IMU data pre-processing, the average time per frame was only approximately 10 ms and no large deviations were observed for the entire process. For LIO, scan matching required approximately 3 ms, and iterative matching required up to 10 ms for convergence. The construction of the local map required approximately 2 ms. Because loop detection requires circular judgment and ICP-based alignment between loop frames for pose graph optimization, the average loop detection time was approximately 35 ms, and the average time for pose graph optimization was approximately 13 ms, which quickly satisfied the convergence condition.

Combined with the system framework diagram (Figure 1), we can see that we tightly couple the 200 hz operating frequency IMU pre-integration with the 10 hz operating frequency LiDAR and synchronize the 10 hz LiDAR-inertial odometry output to the back end optimization module through the tight coupling. While the front end and back end of our constructed LiDAR-IMU fusion SLAM system work as two independent threads, the sum of each frame of data processing time of each front end and back end module can meet the system’s real-time requirements as long as it is less than 100 ms (10 hz).

## 8. Conclusions

(1) A tightly coupled LiDAR-IMU fusion SLAM algorithm for perceptually degraded scenes is proposed. The robustness for such special scenarios is enhanced by constructing an iterative Kalman filter at the front end and optimizing the pose map at the back end. By adding loop detection and ground constraints to the tightly coupled frame, the relative poses between adjacent keyframes are optimized, the cumulative sensor errors are reduced, the accuracy of the system is improved and global map consistency is ensured.
(2) The algorithm was quantitatively and qualitatively analyzed on public and self-selected datasets and compared with existing LiDAR-SLAM algorithms. The results show that the algorithm was able to achieve robust 6DOF state estimation and high-performance SLAM in the test environments and output motion trajectories, global maps and state estimation after pose graph optimization.
(3) Ablation experiments were performed on the algorithm in terms of ground constraint and loop detection to determine their impacts on the entire system and to explain the rationality of the algorithm. The running time of each major module on the front end and back end of the system was statistically analyzed for the different datasets, and the proposed algorithm was able to meet the real-time performance requirements.
(4) In subsequent work, research on multi-sensor fusion based on LiDAR, vision and IMU will be conducted in conjunction with other practical scenarios of perceptual degradation to further improve the positioning accuracy and robustness of the system. In addition, the structure of the stored point cloud map data, which is the basis for the map-based positioning and navigation of the robot, was studied and is the key to ensuring real-time performance.

## Figures and Tables

**Table 1:** Meaning of the variables involved in this paper.

Symbol	Meaning	Symbol	Meaning
$p\in {\mathbb{R}}^{3}$	Position	$p_{A}^{B}$	Translation of *A* to *B* (matrix)
$v\in {\mathbb{R}}^{3}$	Velocity	$q_{A}^{B}$	Rotation of *A* to *B* (quaternion)
$\theta \in {\mathbb{R}}^{3}$	Euler angular pose	$R_{A}^{B}$	Rotation of *A* to *B* (matrix)
q	Quaternion pose	$t_{A}^{B}$	Translation of *A* to *B* (vector)
$T\in {\mathbb{R}}^{4}$	Transformation matrix	$T_{A}^{B}$	Transformation matrix from *A* to *B*
$\omega \in {\mathbb{R}}^{3}$	Angular velocity	w	World coordinates
$a\in {\mathbb{R}}^{3}$	Acceleration	l	LiDAR coordinates
$g\in {\mathbb{R}}^{3}$	Gravity	b	Robotics, IMU coordinates
$b_{a},b_{\omega }\in {\mathbb{R}}^{3}$	Acceleration and angular velocity bias	$b_{k}$	kth frame of LiDAR under *B*
$n_{a},n_{\omega }\in {\mathbb{R}}^{3}$	Acceleration and angular velocity noise	${\left(•\right)}^{k}$	State of the *k*th frame
P	3D point cloud	${\left(•\right)}_{t}$	State at moment *t*
M	Map	$\left(\hat{•}\right)$	Priori state

**Table 2:** Rotation and translation errors for comparing true values in the following six fast-to-slow datasets.

Error	Dataset	LOAM	LIO-Raw	LIO-No-Ext	LIO	Ours
Root mean square error of translation (m)	Fast_1	0.4469	0.2464	0.0957	0.0949	**0.0700**
	Fast_2	0.2023	0.4346	0.1210	0.0755	**0.0683**
	Med_1	0.1740	0.1413	0.1677	0.1002	**0.0577**
	Med_2	0.1010	0.2460	0.3032	0.1308	**0.0647**
	Slow_1	0.0606	0.1014	0.0838	0.0725	**0.0248**
	Slow_2	0.0666	0.1016	0.0868	0.1024	**0.0310**
Root mean square error of rotation (rad)	Fast_1	0.1104	0.1123	0.0547	0.0545	**0.0283**
	Fast_2	0.0763	0.1063	0.0784	0.0581	**0.0277**
	Med_1	0.0724	0.0620	0.0596	0.0570	**0.0198**
	Med_2	0.0617	0.0886	0.0900	0.0557	**0.0287**
	Slow_1	0.0558	0.0672	0.0572	0.0581	**0.0309**
	Slow_2	0.0614	0.0584	0.0571	0.0533	**0.0284**

**Table 3:** Error statistics of relative translations and rotations between the four algorithms and the true values.

Algorithm	Aloam	Lego_Loam	Lio_Sam	Ours	Aloam	Lego_Loam	Lio_Sam	Ours
Error	Translation (m)				Rotation (deg)			
Max	1.2899	2.5120	4.7334	**0.0814**	2.9888	6.3016	3.2062	**0.6162**
Mean	0.1015	1.1715	1.5112	**0.0105**	0.2889	0.9832	0.2777	**0.0381**
Min	0.0032	0.0092	0.0066	**0.0018**	0.0027	0.0073	0.0039	**0.0168**
RMSE	0.1175	1.2414	1.7136	**0.0154**	0.3678	1.5164	0.3667	**0.0641**
Std	0.0591	0.4108	0.8079	**0.0112**	0.2277	1.1545	0.2395	**0.0515**

**Table 4:** Average running time statistics for each major module.

Submodule Name		Self-Picked Datasets (ms)	KITTI Dataset (ms)
Front End	down sampling	4.14	14.13
	pre-processing	2.89	8.68
	scan matching	3.45	16.21
Back End	map construction	2.19	4.74
	loop detection	34.58	125.35
	pose graph optimization	13.41	118.62

## Data Availability

Data available in a publicly accessible repository that does not issue DOIs.
